# Rethinking Right Upper Quadrant (RUQ) Pain: The Role of Lemmel Syndrome in Biliary Obstruction

**DOI:** 10.7759/cureus.76442

**Published:** 2024-12-26

**Authors:** Karan Yagnik, Sai Gaddameedi, Jayasree Ravilla, Payal Chhabria, Malay Rathod, Anoohya Vangala, Doantrang Du, Ben Terrany

**Affiliations:** 1 Internal Medicine, Monmouth Medical Center, Long Branch, USA; 2 Medicine, Monmouth Medical Center, Long Branch, USA; 3 Internal Medicine, RWJBarnabas Health, Long Branch, USA; 4 Gastroenterology and Hepatology, Monmouth Medical Center, Long Branch, USA

**Keywords:** diagnostic and therapeutic ercp, lemmel’s syndrome, percutaneous transhepatic biliary drainage, periampullary duodenal diverticulum, right upper quadrant abdominal pain

## Abstract

Lemmel syndrome involves a periampullary duodenal diverticulum (PAD), a pouch-like outpouching near the ampulla of Vater, compressing the common bile duct. We describe a case of severe abdominal pain in a patient who had a large periampullary diverticulum, managed with surgical intervention after an initial failed endoscopic retrograde cholangiopancreatography (ERCP). An elderly female patient in her early 90s arrived at the emergency department with severe cramping pain localized to the right upper quadrant of her abdomen, progressively intensifying over several weeks. Her blood pressure measured 161/68 mmHg, while other vital signs and the physical exam showed no abnormalities. A CT scan of the chest, abdomen, and pelvis with IV contrast revealed both biliary and pancreatic duct dilation, along with choledocholithiasis and a possible obstructing lesion at the pancreatic head. Further imaging with MRI and MRCP confirmed choledocholithiasis, dilation of the common bile duct (CBD), and the presence of a duodenal diverticulum. The initial attempt at endoscopic retrograde cholangiopancreatography (ERCP) was unsuccessful due to a large periampullary diverticulum, leading to the placement of a temporary percutaneous cholecystostomy tube. In a subsequent ERCP, the stones were successfully removed. During the same hospital stay, she underwent cholecystectomy and was later discharged. Patients experiencing right upper quadrant (RUQ) pain should consider Lemmel syndrome as one of the differential diagnoses. Although rare, it is a treatable condition that, if overlooked, can result in repeated hospitalizations and ongoing investigations. The altered anatomy associated with this syndrome can complicate standard medical procedures, requiring physicians to adapt their approach and utilize alternative methods.

## Introduction

Lemmel syndrome involves a periampullary duodenal diverticulum (PAD), a pouch-like outpouching near the ampulla of Vater, compressing the common bile duct [1,2]. The most common presenting symptom is RUQ pain, such as in our case; however, it can be very non-specific or even entirely asymptomatic at presentation. The onset of symptoms can be acute or subacute and can be associated with obstructive jaundice, although diagnosis is often made in the absence of jaundice, as depicted in our case report [1-3]. Periampullary diverticulum (PAD) is defined as a pseudodiverticulum located within 2-3 centimeters of the ampulla of Vater [3]. At the same time, incidental PAD is found in roughly 22% of the general population, and fewer than 10% of those affected experience any symptoms [4]. Clinical significance arises when PAD presents with complications such as diverticulitis, bleeding, perforation, obstructive jaundice (Lemmel syndrome), choledocholithiasis, pancreatitis, or cholangitis [3].

Most cases are diagnosed through imaging modalities such as CT scans and/or MRIs, especially MRCP (magnetic resonance cholangiopancreatography). Treatment options vary from conservative management to surgical interventions such as excision, stenting, or sphincterotomy, depending on the clinical scenario, with diverticulectomy being the most definitive treatment [5]. We describe a case of severe abdominal pain in a patient who had a large periampullary diverticulum, managed with surgical intervention after an initial failed endoscopic retrograde cholangiopancreatography (ERCP).

*"The abstract of this article was presented to the ACG 2024 annual meeting in Philadelphia, PA, USA, as “A Case of Lemmel's Syndrome: A Rare Cause of Biliary Obstruction in Clinical Practice,' which is being published in the American Journal of Gastroenterology as an abstract."* [6]

## Case presentation

A woman in her early 90s, with a medical background of anemia, breast cancer, hypertension, and high cholesterol, was hospitalized due to severe and persistent abdominal pain. She described the pain as originating in the right upper quadrant, cramping in nature, and progressively intensifying over several weeks, rating it 7 out of 10 on the pain scale upon her arrival. The discomfort was exacerbated by eating and somewhat alleviated with opioid medication. Notably, she denied experiencing any associated symptoms such as nausea, vomiting, fever, chills, or other concerning complaints.

**Figure 1:**
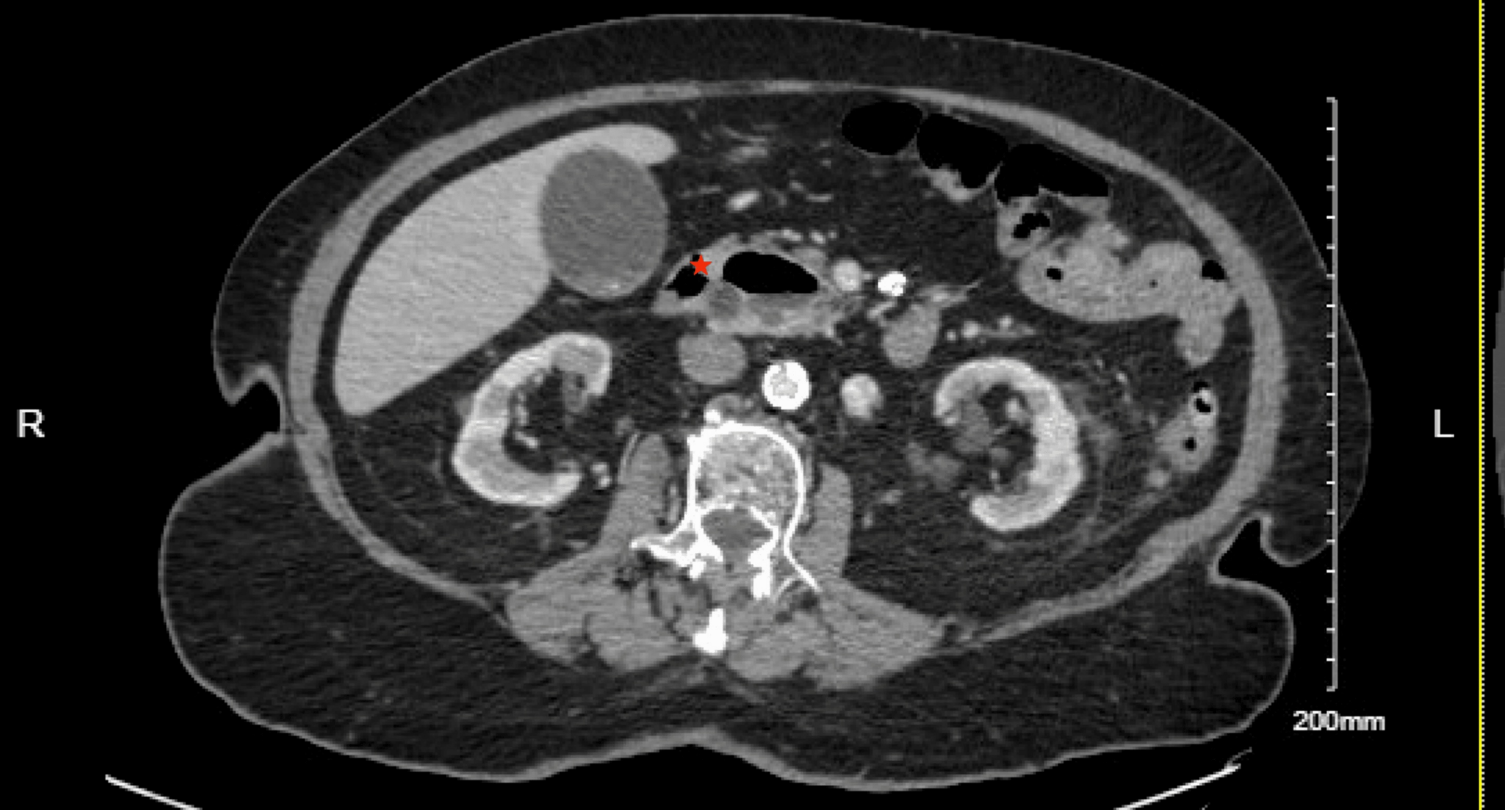
Large periampullary diverticulum at distal common bile duct (CBD) (Red Star) causing CBD dilatation.

**Figure 2:**
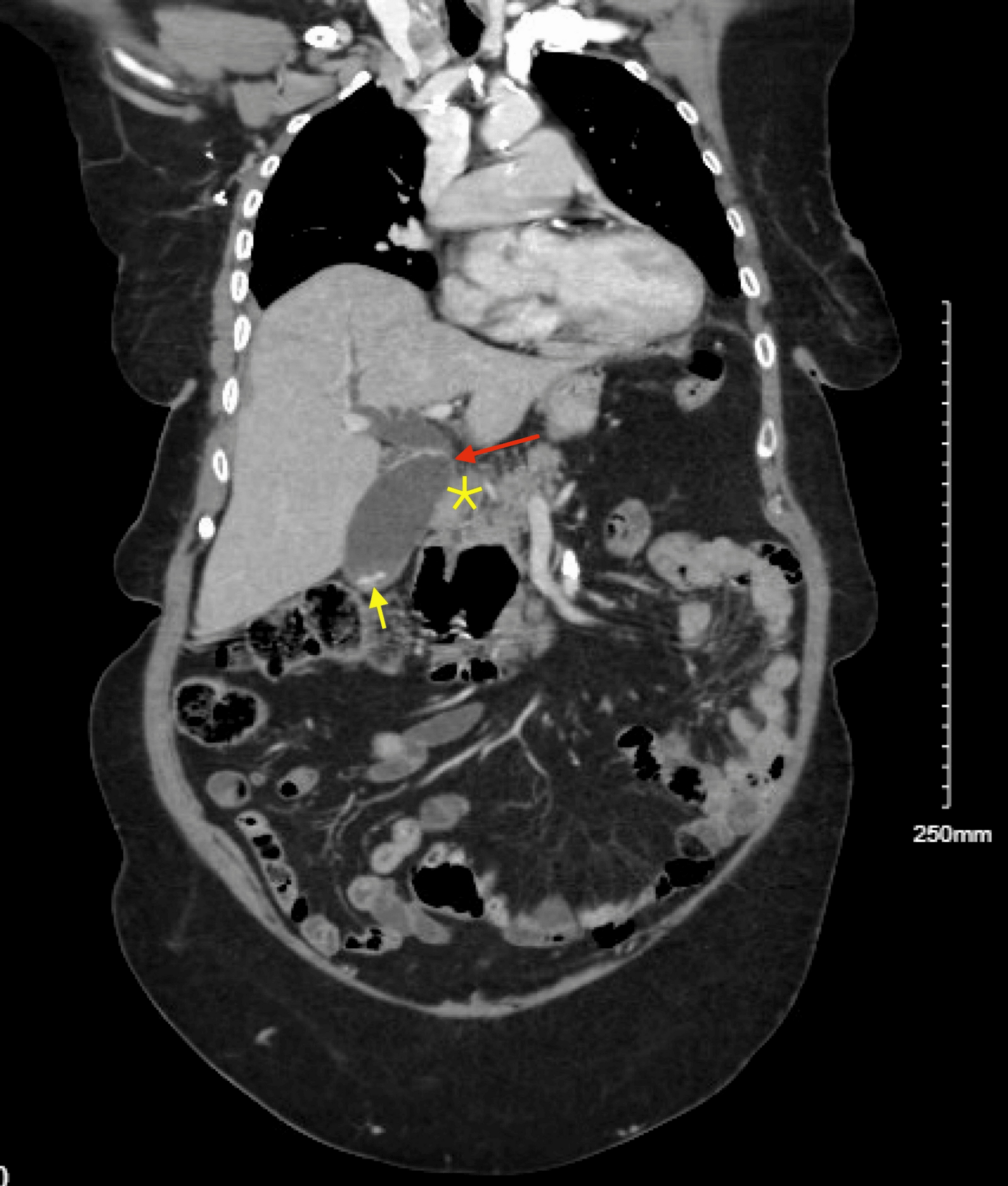
Distended common bile duct and gallbladder (red arrow), choledocholithiasis and cholelithiasis (yellow arrow), and possible obstruction at pancreatic head (yellow star).

Upon arrival, the patient’s vital signs were stable, with a blood pressure of 161/68 mmHg, a heart rate of 78 beats per minute, a respiratory rate of 18 breaths per minute, and normal oxygen saturation on room air. She was afebrile, and her physical examination was largely unremarkable, with no abdominal tenderness noted. Labs were significant for elevated ALT 175 U/L and AST 134 U/L on presentation; alkaline phosphatase (77 U/L) and total bilirubin (0.3 mg/dL) were within the normal limit. A CT scan of the chest, abdomen, and pelvis with intravenous contrast showed dilation of the biliary and pancreatic ducts, along with findings of choledocholithiasis and a possible obstructive lesion at the head of the pancreas. A large duodenal diverticulum was also observed, encasing the distal portion of the common bile duct and suggesting biliary obstruction (Figures 1, 2). Follow-up imaging with MRI and MRCP confirmed dilation of both intrahepatic and extrahepatic bile ducts, with several filling defects in the common bile duct, consistent with choledocholithiasis, as well as CBD dilation and the presence of a duodenal diverticulum.

The first attempt at endoscopic retrograde cholangiopancreatography (ERCP) was unsuccessful because a large periampullary diverticulum obstructed access, complicating the cannulation of the common bile duct (CBD) and hindering injection. Consequently, interventional radiology (IR) placed a temporary percutaneous cholecystostomy tube to relieve the biliary obstruction. The patient was then transferred to our facility for further management. Subsequently, a repeat ERCP was performed with sphincterotomy, successfully removing all stones from the CBD. ALT and AST normalized after successful stone removal and biliary drainage. During the same hospital stay, the patient successfully underwent a cholecystectomy and, following an uncomplicated recovery, was discharged in stable condition.

## Discussion

A duodenal diverticulum is categorized as periampullary, especially when it arises within 2-3 cm of the ampulla of Vater [7]. Periampullary diverticulum (PAD) causes ampullary dysfunction either due to functional dysfunction, persistent inflammation leading to fibrosis, or mechanical compression of the distal common bile duct and ampulla [7-10]. Diagnosis of Lemmel syndrome is made by imaging. The cholestatic pattern of serum LFTs (elevated alkaline phosphatase (ALP), direct bilirubin, and/or transaminase) can be supportive, but it is not uncommon in normal serum chemistry despite radiographic evidence of Lemmel syndrome, such as depicted in our case where ALT and AST were elevated but ALP and bilirubin were within normal limits [1,11]. There is no standardized approach for imaging; ultrasonography is usually used as the first choice. Interestingly, diagnosis of Lemmel syndrome has never been made only with ultrasound; an additional mode of imaging is usually required. A CT scan of the abdomen and pelvis with intravenous contrast is sensitive and commonly performed, often being sufficient for diagnosis. However, the lack of specificity of CT scans is often deceptive as pancreatic neoplasms or pseudocysts [11,12,7]. Therefore, more specific modalities such as magnetic resonance cholangiopancreatography (MRCP) or endoscopic ultrasound are often necessary. ERCP comes in a little more handy as it offers both diagnostic and therapeutic benefits; however, diagnosis can be made only 50% of the time with the endoscopy, hence imaging such as MRCP still needs to be performed [1,11,13].

There is no standardized treatment approach, and clinical decisions should be tailored to each individual patient, and the most definitive treatment is diverticulectomy [13]. However, not all patients warranted surgical intervention. Asymptomatic or minimally symptomatic patients can be managed conservatively with a high-fiber diet as an option [1,2,7,14]. Patients with obstructive pancreaticobiliary complications warrant interventional approaches such as ERCP with sphincterotomy or stenting, diverticulectomy with or without antibiotic coverage (antibiotic use is necessary in the setting of cholangitis, sepsis, and such as in high-risk patients) [1,9]. However, many cases have been reported as unique approaches varying upon a subjective basis, such as diverticular lavage as a bridge for diverticulectomy for symptomatic relief, and extracorporeal shock wave lithotripsy [14,15]. Altered anatomy in such uncommon cases can make it challenging to perform usual procedures. In our case, we initially attempted an ERCP, but after a failed attempt, interventional radiology performed a percutaneous cholecystostomy to prevent sepsis from choledocholithiasis. Eventually, we conducted an ERCP with sphincterotomy for the drainage of sludge and stones and to relieve compression.

In our case, while CBD (common bile duct) stones are likely contributing to abdominal pain, we cannot entirely dismiss the possibility that compression from a periampullary diverticulum (PAD) is playing a role. The compression could cause the pain directly or indirectly, leading to cholestasis, which may have facilitated stone formation. Additionally, it was unexpected that the patient's alkaline phosphatase (ALP) and bilirubin levels remained normal in the context of choledocholithiasis, despite elevated ALT and AST levels. However, the normalization of aspartate aminotransferase (AST) and alanine aminotransferase (ALT) levels following sphincterotomy highlights the combined influence of both the diverticulum and the stone on this clinical presentation. This suggests that the interaction between these two factors, diverticular compression and the presence of stones, was likely responsible for the symptoms and abnormal liver function tests observed before the procedure.

## Conclusions

In summary, the diagnosis of Lemmel syndrome should be considered when there is evidence of compression or dilation of the pancreatic duct and/or common bile duct, whether or not cholestasis is present. This diagnosis is typically made using imaging modalities, with or without supporting endoscopic findings. In our case, the diagnosis was established through a CT scan. However, in more subtle cases of biliary obstruction, especially when stones are present or there is a potential obstructing lesion at the head of the pancreas, MRCP may be required to confirm the diagnosis. Treatment approaches for Lemmel syndrome vary based on symptom severity. Patients with mild or no symptoms can often be managed conservatively with dietary modifications and, in some cases, diverticular lavage. For more severe presentations, invasive treatments may be necessary, with diverticulectomy being the definitive solution for those with significant or refractory symptoms.

Through this case, we aim to contribute to the limited body of literature on Lemmel syndrome, underscoring the importance of recognizing it as a potential, albeit rare, cause of right upper quadrant (RUQ) pain. Failure to diagnose Lemmel syndrome can lead to recurrent hospitalizations and unnecessary investigations, making early recognition critical in preventing repeated diagnostic delays and interventions. Altered anatomy can complicate standard procedures, prompting physicians to adapt by using alternative techniques to ensure effective treatment and safety.
